# Supplementary material for: GDE7 produces cyclic phosphatidic acid in the ER lumen functioning as a lysophospholipid mediator
Source: Commun Biol. 2023 May 16;6:524. doi: 10.1038/s42003-023-04900-4 (PMC10188492; doi:10.1038/s42003-023-04900-4)
Supplement: Supplementary file 2 — Supplementary Information [file 42003_2023_4900_MOESM2_ESM.pdf]

Supplementary information

GDE7 produces cyclic phosphatidic acid in the ER lumen functioning as a lysophospholipid mediator

Keisuke Kitakaze [1, #], Hanif Ali [2], Raiki Kimoto [1, 3], Yasuhiro Takenouchi [1], Hironobu Ishimaru [1], Atsushi Yamashita [4], Natsuo Ueda [5], Tamotsu Tanaka [2], Yasuo Okamoto [1], Kazuhito Tsuboi [1, #]

1 Department of Pharmacology, Kawasaki Medical School, Kurashiki, Okayama, Japan
2 Graduate School of Technology, Industrial and Social Sciences, Tokushima University, Tokushima, Japan
3 Nara Medical University, Kashihara, Nara, Japan
4 Laboratory of Biological Chemistry, Faculty of Pharma-Science, Teikyo University, Tokyo, Japan
5 Department of Biochemistry, Kagawa University School of Medicine, Miki, Kagawa, Japan

# These authors jointly supervised this work

Keisuke Kitakaze, Ph.D.
Department of Pharmacology, Kawasaki Medical School
577 Matsushima, Kurashiki, Okayama 701-0192, Japan
Phone: +81-86-462-1111
E-mail: kitakaze@med.kawasaki-m.ac.jp

Kazuhito Tsuboi, Ph.D.
Department of Pharmacology, Kawasaki Medical School
577 Matsushima, Kurashiki, Okayama 701-0192, Japan
Phone: +81-86-462-1111
E-mail: ktsuboi@med.kawasaki-m.ac.jp

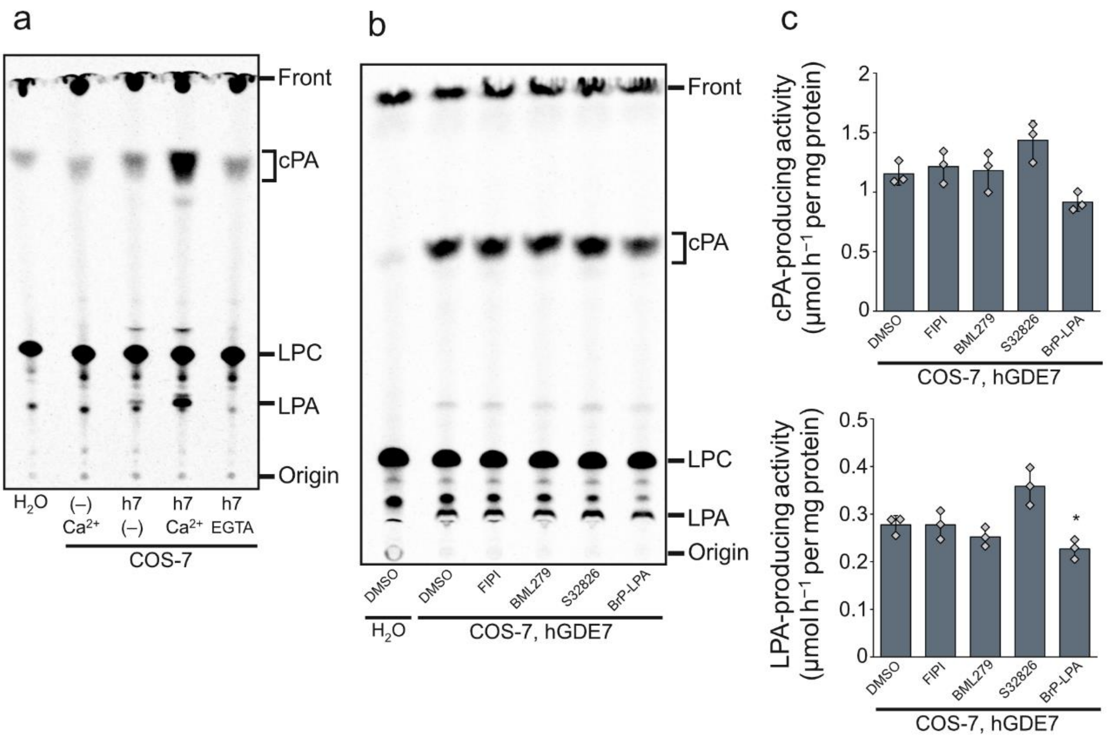

Supplementary Figure 1. cPA-producing activity of hGDE7 in the absence of Ca^{2+} or in the presence of PLD2/ATX inhibitors.

The membrane fractions of COS-7 cells overexpressing hGDE7 (h7) and control cells (–) were incubated with 25 μM 1-[^{14}C]oleoyl LPC for 30 min at 37°C. Radiolabeled lipids were then extracted and separated by TLC. The positions of the origin, LPA, LPC, cPA, and the solvent front on the TLC plates are indicated. Uncropped TLC images are shown in Supplementary Figure 10.

a GDE7 activities were evaluated in the presence of either 2 mM $CaCl_2$ or 2 mM EGTA, or in their absence (–).

b GDE7 activities were evaluated in the presence of 1 μM FIPI (PLD1/2 inhibitor), 1 μM BML279 (PLD1/2 inhibitor), 5 μM S32826 (ATX inhibitor), or 5 μM BrP-LPA (ATX/GDE4/GDE7 inhibitor), or in their absence (DMSO, 0.2%).

c LPA- and cPA-producing activities are shown (mean values ± S.D., n = 3). Dunnett's test was used for analysis. *$P < 0.05$ (vs. DMSO).

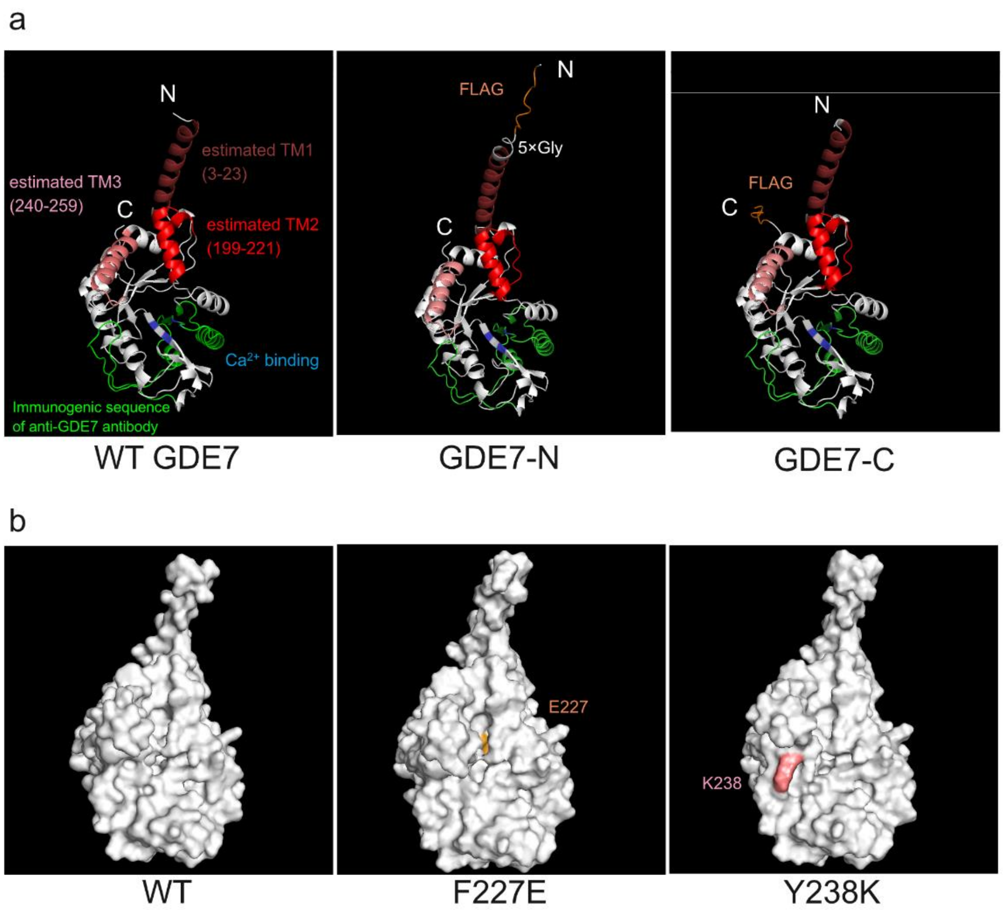

Supplementary Figure 2. Models of hGDE7 proteins.

a The predicted conformation of wild-type (WT) as well as N- and C-terminally FLAG-tagged hGDE7.

b The predicted conformation of hGDE7 mutants, F227E and Y238K.

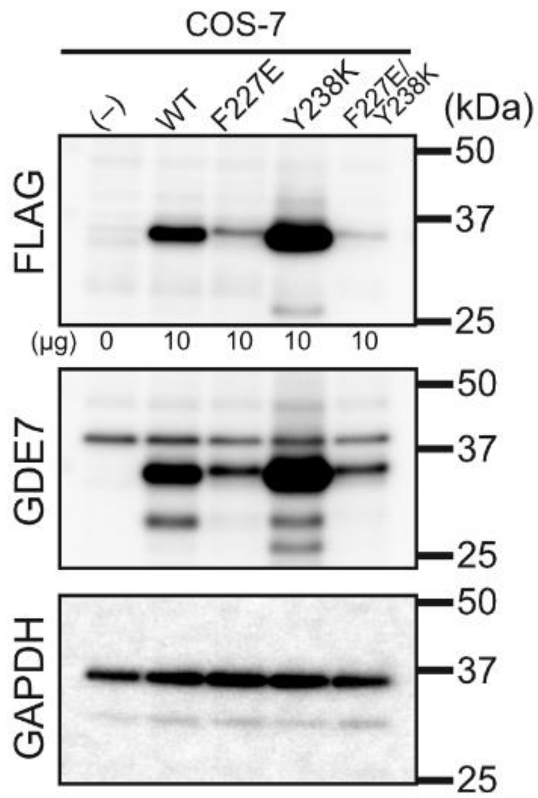

Supplementary Figure 3. GDE7 expression without adjusting protein levels.

The membrane fractions (10 μg) used in Fig. 5b were subjected to immunoblotting with anti-FLAG and anti-GDE7 antibodies. Anti-GAPDH antibody was used as a loading control. Different blots were used for each antibody. Uncropped immunoblot are shown in Supplementary Figure 11.

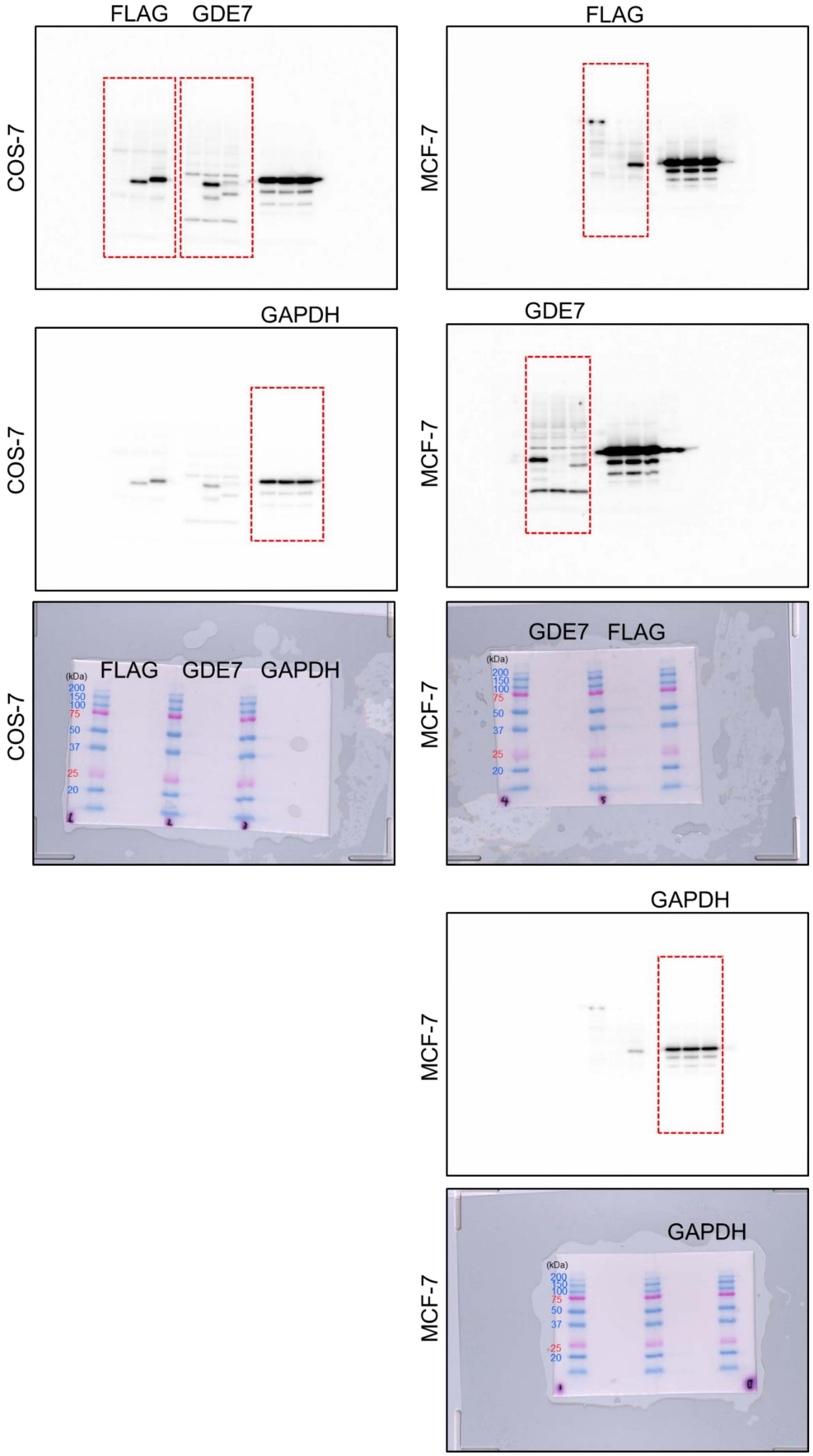

Supplementary Figure 4. Uncropped immunoblots for Fig. 1b and f.

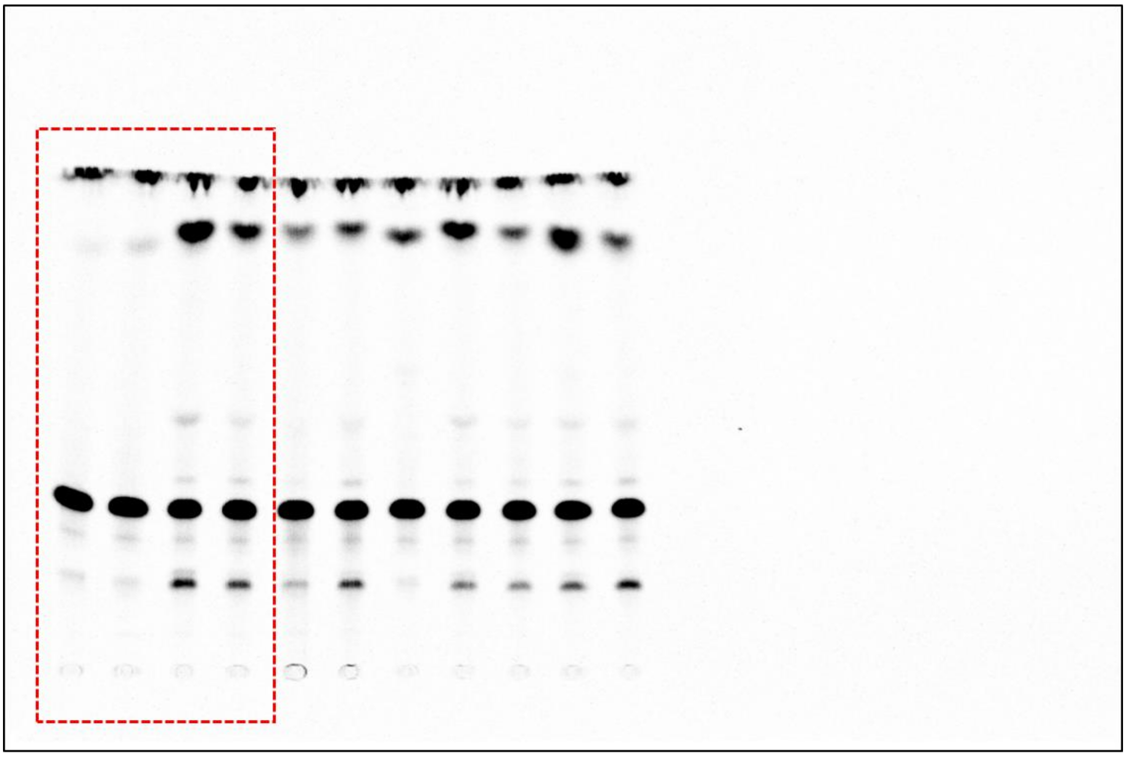

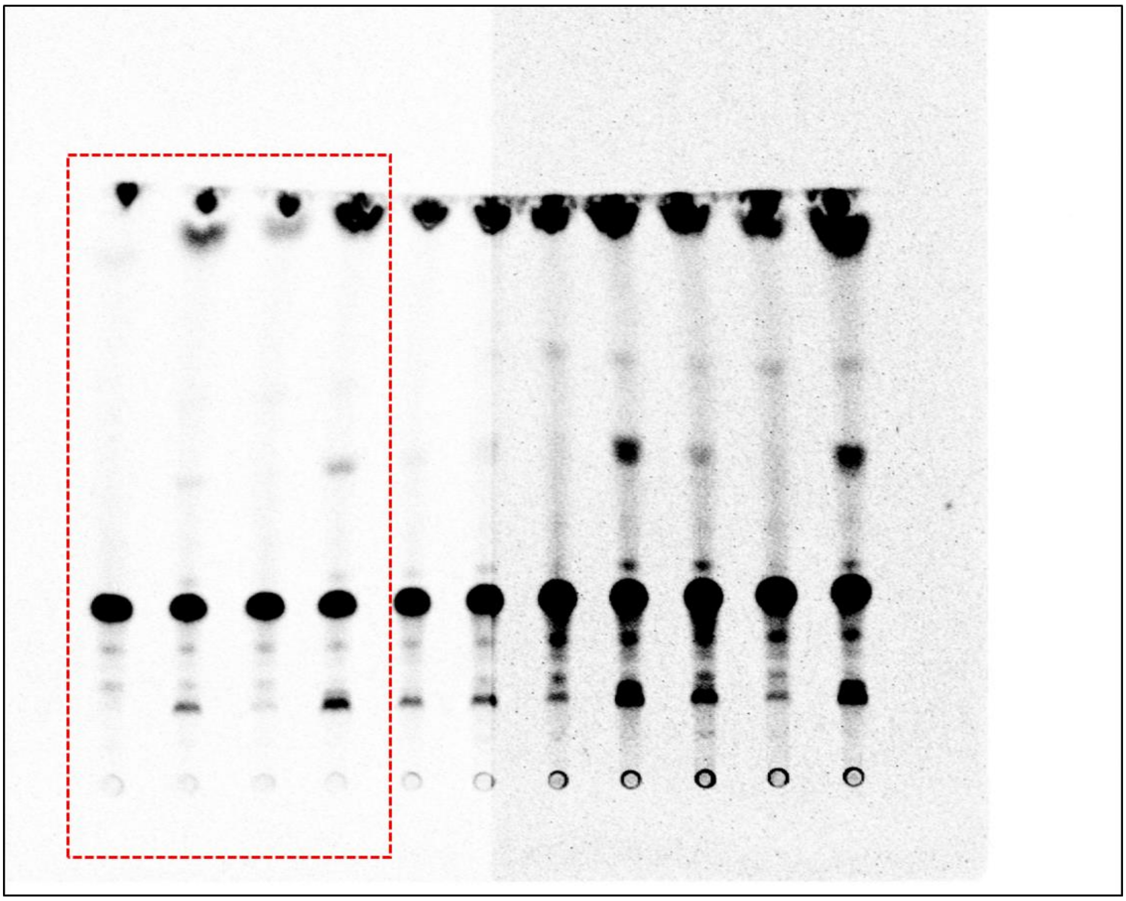

Supplementary Figure 5. Uncropped TLC results for Fig. 1c and g.

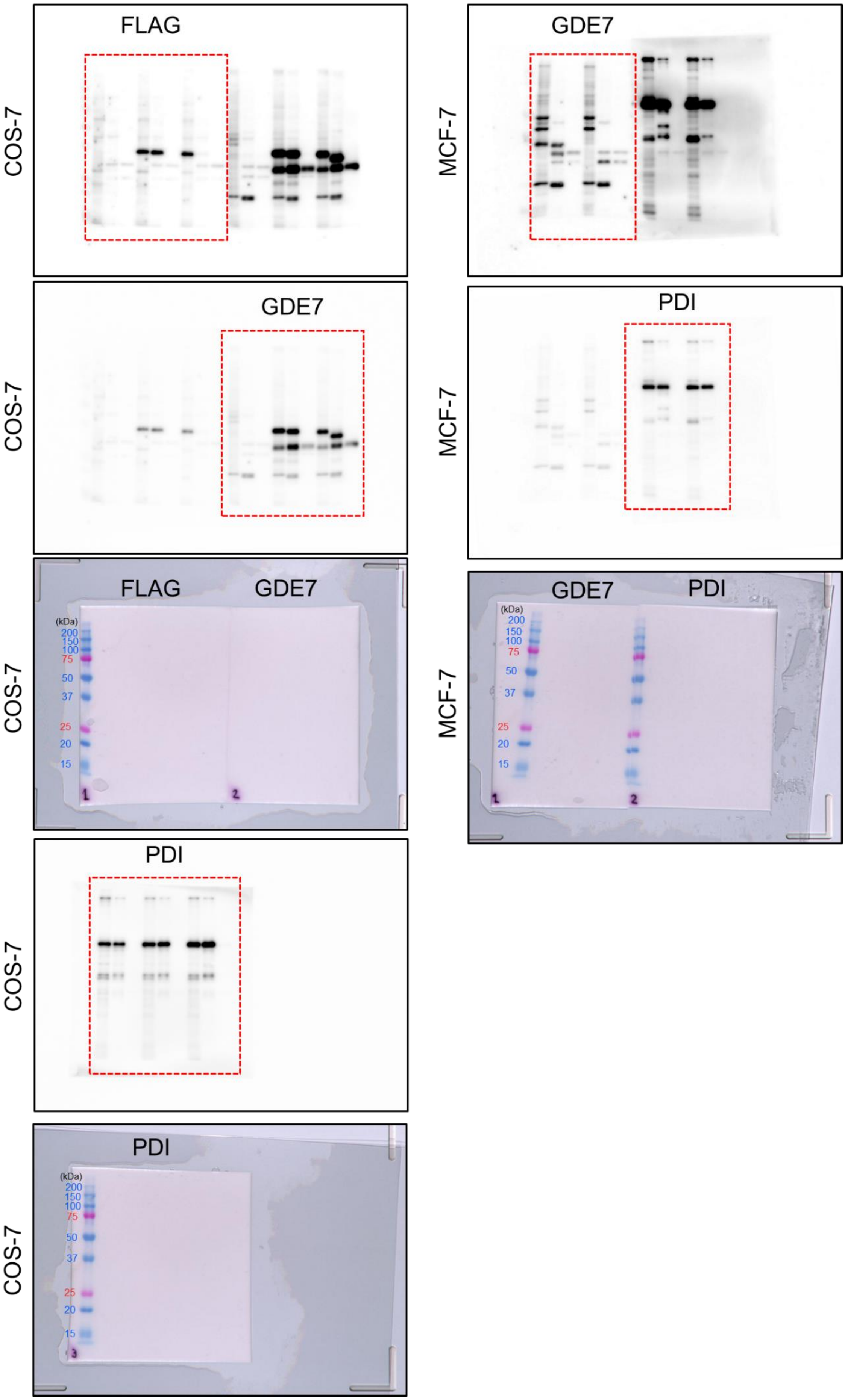

Supplementary Figure 6. Uncropped immunoblots for Fig. 4a and b.

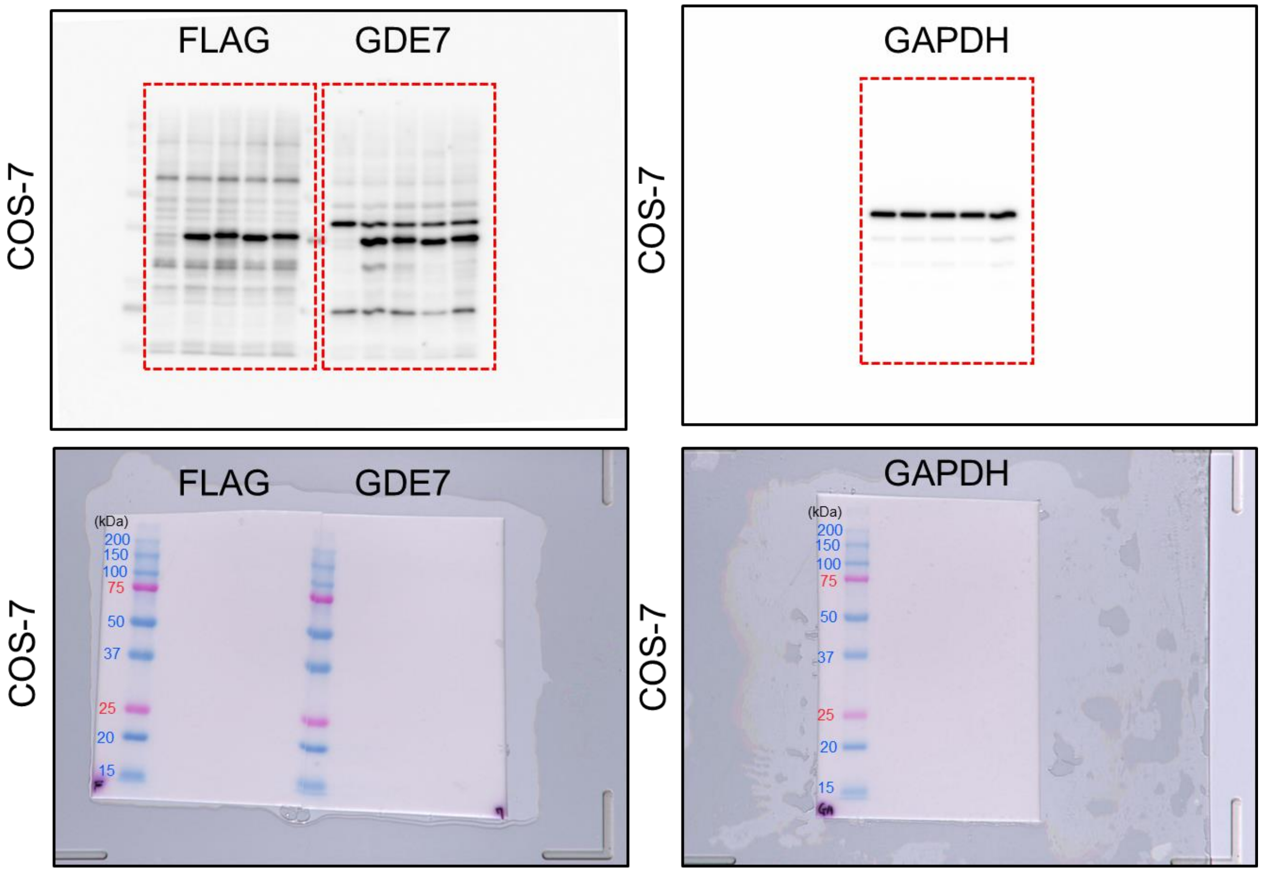

Supplementary Figure 7. Uncropped immunoblots for Fig. 5b.

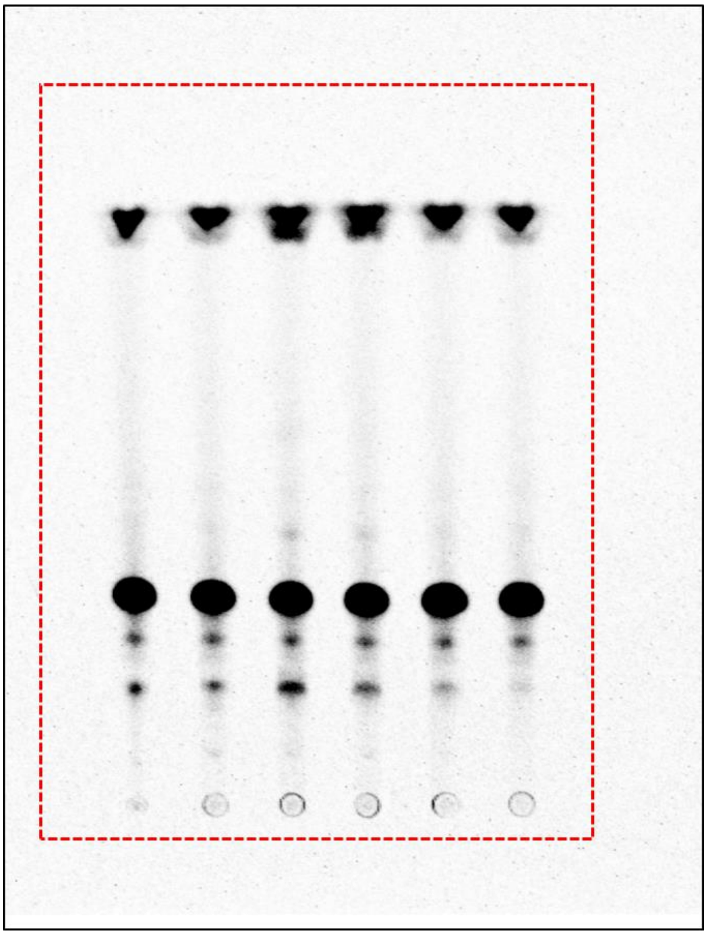

Supplementary Figure 8. Uncropped TLC results for Fig. 5d.

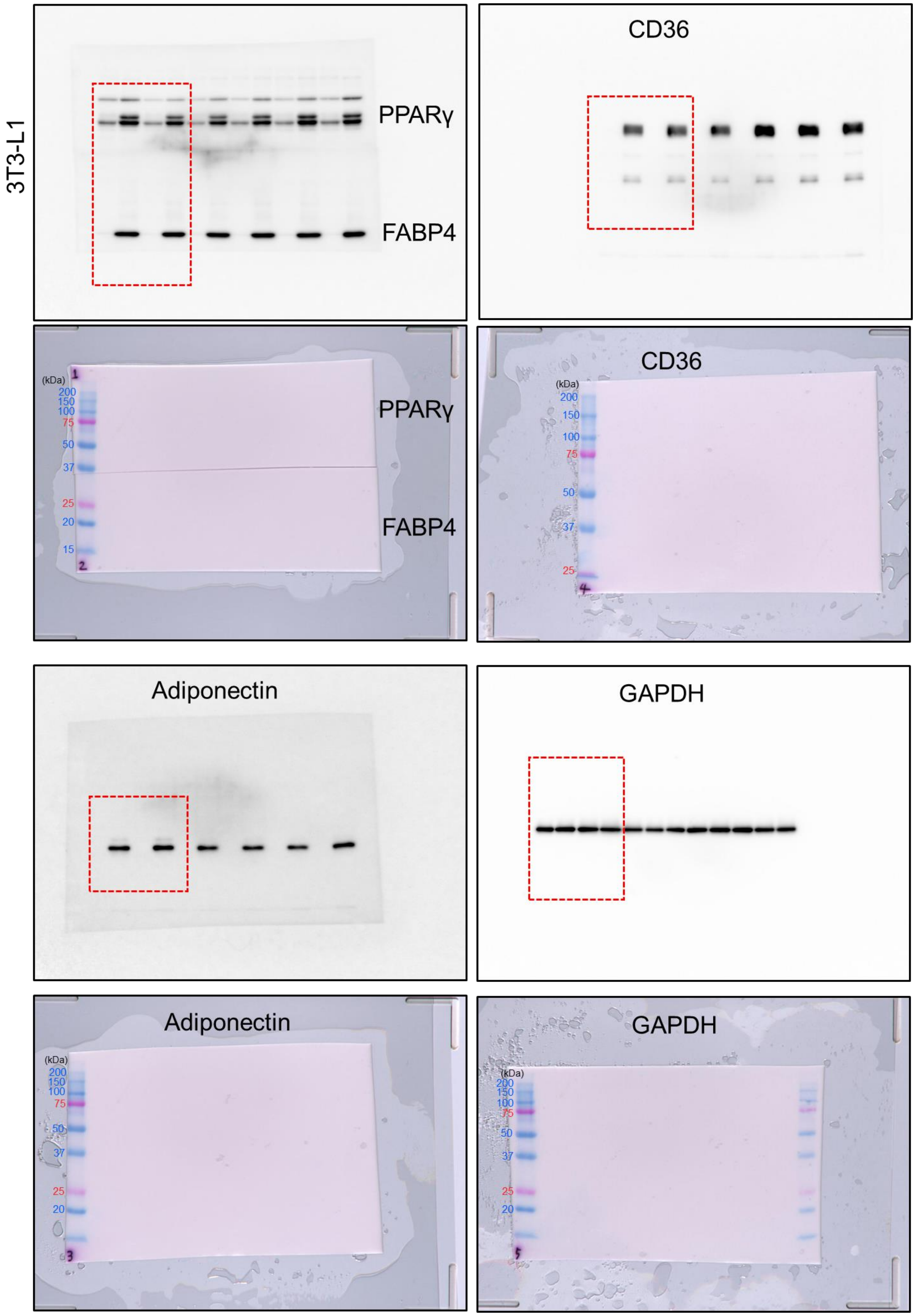

Supplementary Figure 9. Uncropped immunoblots for Fig. 6c.

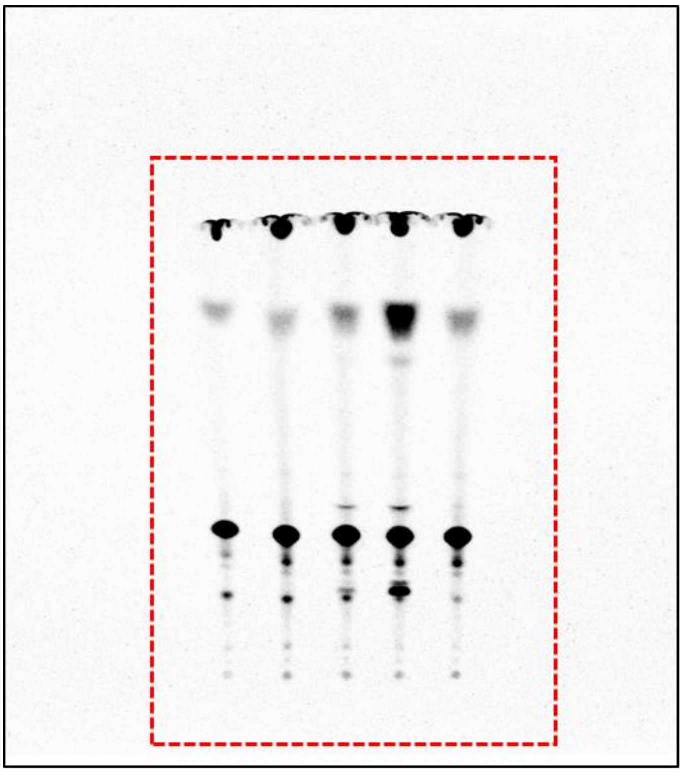

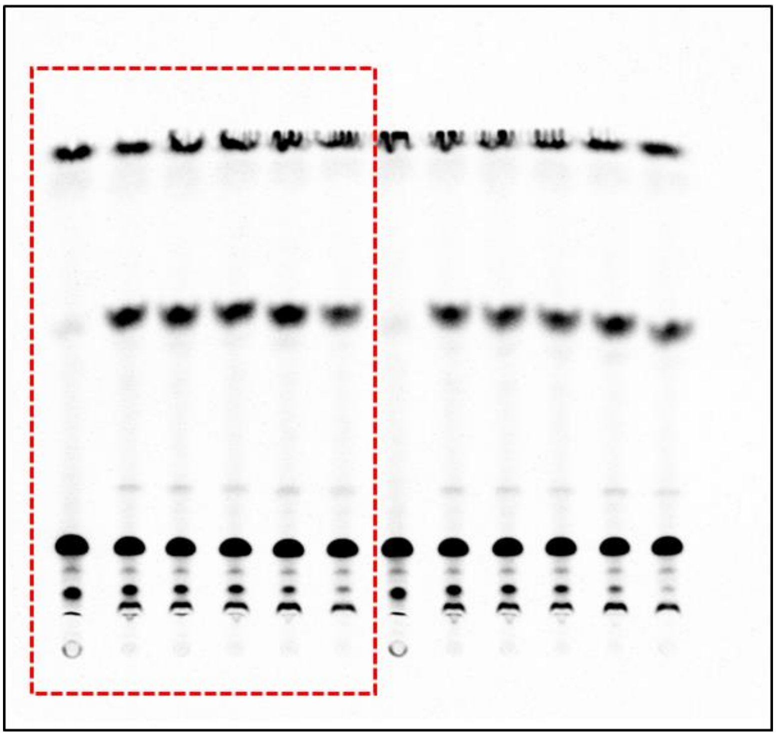

Supplementary Figure 10. Uncropped immunoblots for Supplementary Figure 1a and b.

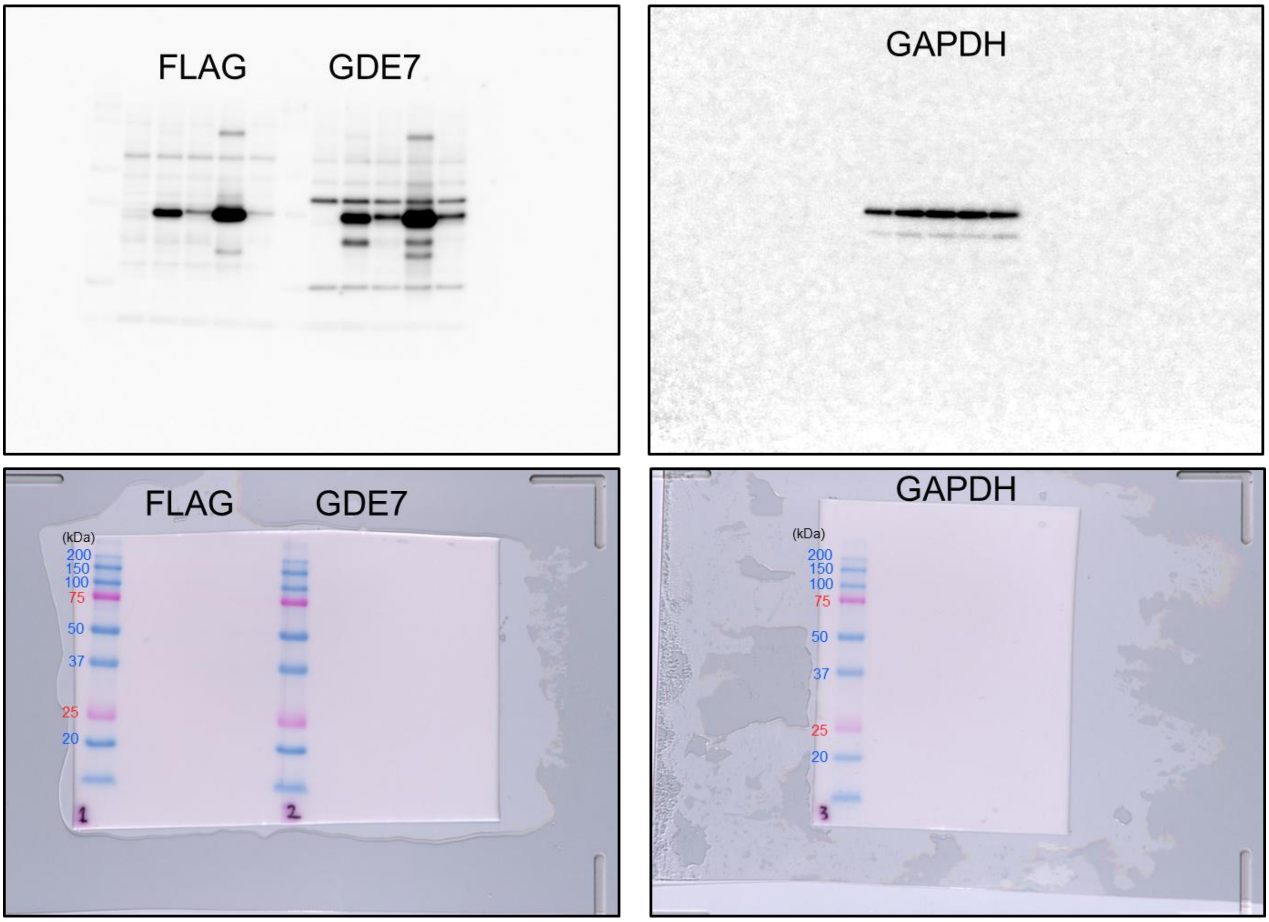

Supplementary Figure 11. Uncropped immunoblots for Supplementary Figure 3.

Supplementary Table 1. Sequences of primers

Name	Application	Sequence (5′–3′)	
		Forward	Reverse
N-terminally FLAG-tagged GDE7	Cloning	GGTCTAGAGCCACCATGGACTACAAAGACGATGACGATAAAGGTGGAGGTGGAGGTAGCCTTTTGCTGTACTATG	AAGGATCCTTAGGAGGTCCGGGCAGCTGGTCCA
F227E (flagment1)	Cloning	GGGACAGCAGAGATCCAGTT	GAAGCATTCGAAGAACTTCTCAGGGATTG
F227E (flagment2)	Cloning	TTCTTCGAATGCTTCCTGCCCAACATCAT	CCCGTTAACTTACTACTTGTCATCGTCAT
Y238K (flagment1)	Cloning	GGGACAGCAGAGATCCAGTT	TGGGAATTTGGTCCTGTTGATGATGTTGG
Y238K (flagment2)	Cloning	AGGACCAAATTCCCATTTTCCTGCTCTTG	CCCGTTAACTTACTACTTGTCATCGTCAT
hCD36	qPCR	TGTCCTGGCTGTGTTTGGAG	CCTTCTTCGAGGACAACTTGC
hCYP27A1	qPCR	GAGGGCAAGTACCCAGTACG	GGTACCAGTGGTGTCCTTCC
hPPARG	qPCR	TGCGAAAGCCTTTTGGTGAC	GGGCTTGTAGCAGGTTGTCT
hGAPDH	qPCR	GGAGCGAGATCCCTCCAAAAT	GGCTGTTGTCATACTTCTCATGG
mCd36	qPCR	GAGTTGGCGAGAAAACCAGTG	GAGAATGCCTCCAAACACAGC
mCyp27a1	qPCR	GGCACCTTTCCTGAGCTG	CACCAGTCACTTCCTTGTGC
mGdpd3	qPCR	TGATCCGACACTTGCAGGAC	GCTGTGGGGTAATCGGTCAT
hGDPD3	qPCR	TAAACAGGGATGTGGGCAGC	TCTGACCCGTGAGCAAAGTG
mAdipoq	qPCR	AGATGGCACTCCTGGAGAGAA	TTCTCCAGGCTCTCCTTTCCT
mFabp4	qPCR	AAACACCGAGATTTCCTTCAAA	TCACGCCTTTCATAACACATTC
mPparg	qPCR	AGGGCGATCTTGACAGGAA	CACCTCTTTGCTCTGCTCCT
mGapdh	qPCR	TGCGACTTCAACAGCAACTC	CTTGCTCAGTGTCCTTGCTG
